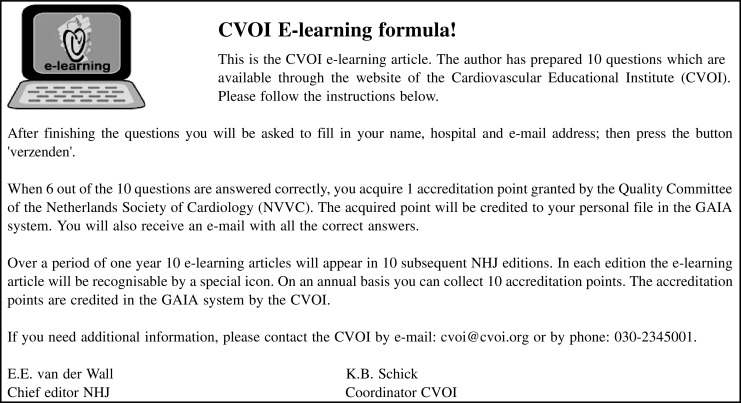# Plasma levels of alpha-1-antichymotrypsin are elevated in patients with chronic heart failure, but are of limited prognostic value

**DOI:** 10.1007/s12471-014-0584-2

**Published:** 2014-08-30

**Authors:** S. I. Lok, D. J. Lok, P. van der Weide, B. Winkens, P. W. Bruggink-André de la Porte, P. A. Doevendans, R. A. de Weger, P. van der Meer, N. de Jonge

**Affiliations:** 1 Department of Cardiology, University Medical Center, Huispostnummer H04.312, PO Box 85500, 3508 GA Utrecht, the Netherlands; 2 Department of Cardiology, Deventer Hospital, Deventer, the Netherlands; 3 Department of Pathology, University Medical Center, Utrecht, the Netherlands; 4 Department of Methodology and Statistics, University of Maastricht, Maastricht, the Netherlands; 5 Department of Cardiology, University Medical Center Groningen, Groningen, the Netherlands

**Keywords:** ACT, Heart failure, Survival

## Abstract

**Background:**

There is increasing interest in utilising novel markers of cardiovascular disease risk in patients with chronic heart failure (HF). Recently, it was shown that alpha-1-antichymotrypsin (ACT), an acute-phase protein and major inhibitor of cathpesin G, plays a role in the pathophysiology of HF and may serve as a marker for myocardial distress.

**Objective:**

To assess whether ACT is independently associated with long-term mortality in chronic HF patients.

**Methods:**

ACT plasma levels were categorised into quartiles. Survival times were analysed using Kaplan-Meier curves and Cox proportional hazards regression, without and with correction for clinically relevant risk factors, including sex, age, duration of HF, kidney function (MDRD), ischaemic HF aetiology and NT-proBNP.

**Results:**

Twenty healthy individuals and 224 patients (mean age 71 years, 72 % male, median HF duration 1.6 years) with chronic HF were included. In total, 159 (71 %) patients died. The median survival time was 5.3 (95 % CI 4.5–6.1) years. ACT was significantly elevated in patients (median 433 μg/ml, IQR 279–680) in comparison with controls (median 214 μg/ml, IQR 166–271; *p* < 0.001). Cox regression analysis demonstrated that ACT was not independently related to long-term mortality in chronic HF patients (crude HR = 1.03, 95 % CI 0.75–1.41, *p* = 0.871; adjusted HR = 1.12, 95 % CI 0.78–1.60, *p* = 0.552), which was confirmed by Kaplan-Meier curves.

**Conclusion:**

ACT levels are elevated in chronic HF patients, but no independent association with long-term mortality can be established.

## Introduction

Despite recent treatment advances, chronic heart failure (HF) continues to impose a substantial healthcare burden. B-type natriuretic peptide (BNP) and the biologically inactive N-terminal fragment (NT-proBNP) are synthesised by ventricular myocytes in response to haemodynamic stress [1]. Natriuretic peptides (NPs) are useful in determining the diagnosis and prognosis of congestive HF and their use is subsequently advocated by the American College of Cardiology [2] and the European Society of Cardiology guidelines [3]. However, NPs have limitations that affect the interpretation of the results. Elevated NP levels can also be seen in the setting of sepsis [4], acute pulmonary embolism [5] and renal dysfunction [6]. NP levels are higher in women than in men and increase with age [7]. Moreover, NP levels may be reduced in obese patients [8, 9]. Consequently, novel biomarkers are currently under intensive investigation and may be of help to improve the prognostication and clinical outcome of HF patients. Recently, we proposed a role of the acute-phase protein alpha-1-antichymotrypsin (ACT; also known as SERPINA3) in reverse remodelling [10]. Our previous data demonstrated that high ACT plasma and myocardial levels in HF decrease during mechanical support. The goal of this study was to evaluate the prognostic role of ACT levels with respect to long-term mortality in chronic HF patients.

## Methods and materials

### Study population

Patient material consisted of plasma and data obtained from the Deventer-Alkmaar Heart Failure study (DEAL-HF) [11, 12]. Briefly, 240 patients with typical signs and symptoms of HF were included, combined with echocardiographic or radionuclide ventriculographic findings of a reduced left ventricular systolic function (LVEF ≤ 45 %) or diastolic dysfunction. The main exclusion criteria were an expected survival of less than 1 year and planned hospitalisation. In the present study, a complete set of data was available for 224 patients at baseline (due to missing blood samples). Control plasma was collected from 20 anonymous healthy individuals. The study was approved by the local Medical Ethics Committees and complied with the *Declaration of Helsinki*. All patients gave written informed consent.

### Laboratory assessment

Routine laboratory measurements and blood samples were obtained at baseline. EDTA plasma was separated and stored at minus 70 °C. Circulating levels of ACT were analysed according to the description of the manufacturer (Genway Biotech Inc, San Diego, USA). In short, the samples were diluted 1:5000. Standards and samples were added in duplicate in a 96-well plate coated with antibody and incubated. After the first washing step, the conjugate was added, followed by incubation. The next washing step was followed by the addition of the substrate solution and incubation. The stop solution was added and wells were read out on a microplate reader.

### Clinical follow-up

ACT values were assessed for all-cause mortality. Patients were followed up to 10.5 years after randomisation at the outpatient clinic. In case of no show, information regarding survival was obtained from the hospital system, relatives or general practitioner.

### Statistics

Categorical data are presented by number (%) and numerical data by mean ± standard deviation or by median (interquartile range, IQR, i.e. 25th–75th percentile), where appropriate. Comparisons between patients and healthy controls were performed using independent-samples *t*-test or Mann-Whitey *U*-test for numerical variables and Chi-square or Fisher’s exact test for categorical variables. Linear regression analysis was performed to assess clinically relevant factors independently related to ACT plasma levels, such as sex, age, duration of HF, kidney function (MDRD), ischaemic HF aetiology and NT-proBNP. Survival times were analysed using Kaplan-Meier curves and Cox proportional hazards (PH) regression, without and with correction for the above-mentioned clinically relevant risk factors. Time to event was defined as time between inclusion and death or to end of study/loss to follow-up (censored). PH assumption was checked using Schoenfeld residuals and linearity assumption by adding and testing mean-centred quadratic terms. A *p*-value ≤0.05 was considered to be statistically significant. All analyses were done with SPSS 20.0 software (SPSS Inc, Chicago, IL).

## Results

### Baseline characteristics

Characteristics of the study population are described in Table 1. The study cohort consisted of patients with severe chronic HF with a mean age of 71 years, 72 % were male with a median HF duration of 1.6 years. Almost all patients (97 %) had left ventricular systolic dysfunction with a reduced ejection fraction, the mean ejection fraction being 31 %. At the time of inclusion, ischaemic aetiology of HF was present in 146 patients (65 %). Non-survivors were older, male subjects with a longer duration of HF, higher C-reactive protein and NT-proBNP levels, more often had kidney dysfunction, were more often diagnosed with diabetes mellitus, anaemia and ischaemic heart disease and were less often treated with beta-blocking agents in comparison with survivors.

**Table 1:** Baseline characteristics

Variable	Total	Survivors	Non-survivors	*p*-value^1^
	(*n* = 224)	(*n* = 65)	(*n* = 159)	
Age, years	71 ± 10	67 ± 11	72 ± 9	0.001
Sex, male (%)	72	60	77	0.008
HF, ischaemic aetiology (%)	65	51	72	0.004
Duration of HF, years	1.6 (0.3–5.8)	0.4 (0.2–3.3)	2.6 (0.4–6.5)	<0.001
NYHA class, III/IV (%)	99	98	99	0.986
Comorbidities				
Diabetes (%)	30	20	33	0.047
COPD (%)	28	29	27	0.740
CVA (%)	10	8	11	0.417
Hypercholesterolaemia (%)	47	48	49	0.853
Anaemia (%)	17	8	20	0.023
Laboratory				
Haemoglobin, mmol/l	8.4 ± 1.0	8.6 ± 0.8	8.3 ± 1.0	0.135
Sodium, mmol/l	138 ± 3	139 ± 3	138 ± 3	0.030
Potassium, mmol/l	4.4 ± 0.5	4.4 ± 0.4	4.4 ± 0.5	0.446
Urea, mmol/l	11.0 ± 5.4	9.6 ± 4.1	11.6 ± 5.8	0.005
Creatinine, μmol/l	127 ± 36	115 ± 23	131 ± 40	<0.001
MDRD	52 ± 14	55 ± 13	51 ± 15	0.086
Leukocytes, ×10^9/l	7.8 ± 2.2	7.5 ± 1.8	7.9 ± 2.4	0.157
CRP, mg/l	8 (5–16)	6 (3–11)	10 (6–18)	<0.001
NT-proBNP, pg/ml	2089 (973–4364)	1725 (727–3341)	2360 (1074–5776)	0.003
Medication use				
ACE inhibitors (%)	88	91	87	0.419
ARBs (%)	16	17	15	0.711
Beta-blockers (%)	79	89	75	0.022
Calcium blockers (%)	7	8	6	0.769
Digoxin (%)	28	17	33	0.019
Diuretics (%)	99	98	99	0.988
Nitrates (%)	41	42	40	0.750

Values are presented as means ± standard deviations, medians ± interquartile ranges or as frequencies and percentages

*HF* heart failure, *NYHA* New York Heart Association, *COPD* chronic obstructive pulmonary disease, *CVA* cerebrovascular accident, *CRP* C-reactive protein, *NT-proBNP* N-terminal pro-brain natriuretic peptide, *ACE* angiotensin-converting enzyme, *ARB* angiotension receptor blocker

^1^ comparison survivors versus non-survivors

### Plasma levels of ACT in patients and healthy controls

**Fig. 1:**
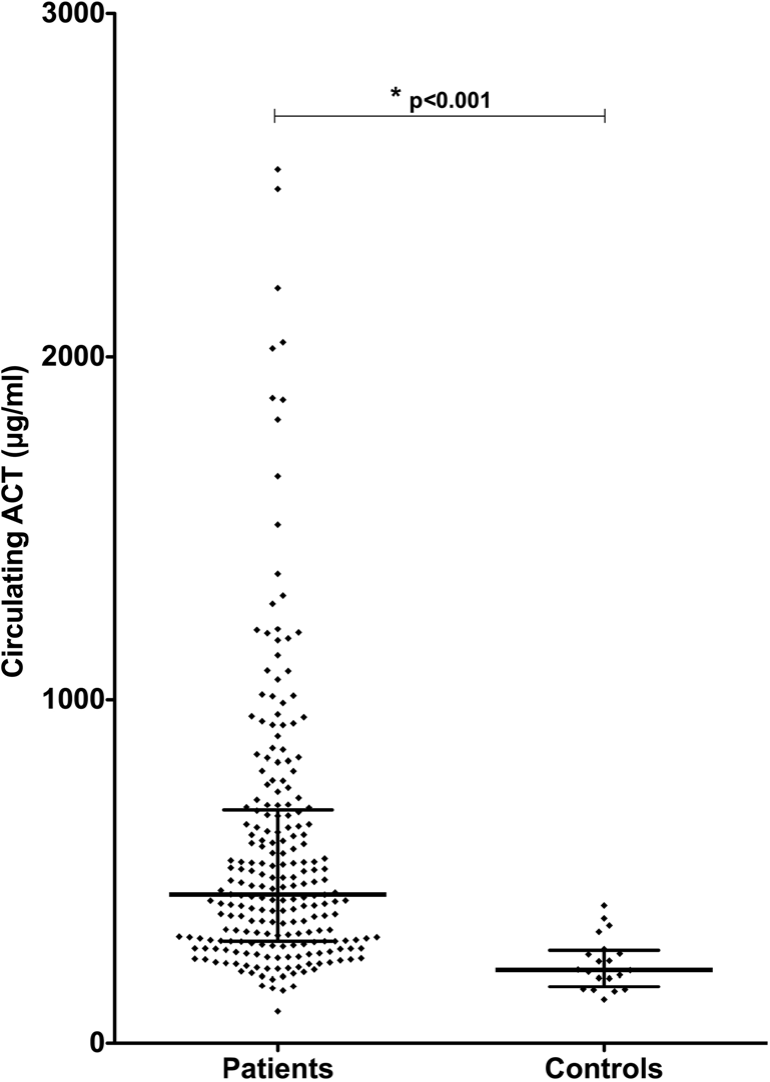
Alpha-1-antichymotrypsin (ACT) plasma levels in patients with chronic heart failure (HF) and healthy controls. ACT levels were significantly higher in chronic HF patients in comparison with controls (*p* < 0.001). Each *dot* represents one patient; the *lines* indicate median and IQR

Figure 1 shows the plasma levels of ACT in patients and healthy controls. A large individual variation of ACT was found. ACT was significantly elevated in patients (median 433 μg/ml, IQR 279–680) in comparison with controls (median 214 μg/ml, IQR 166–271; *p* < 0.001). A linear regression analysis showed that the duration of HF was independently related to ACT plasma levels (patients with a shorter duration of HF had a higher ACT plasma level; *p* = 0.031). Only 5.4 % of the variation in ACT plasma levels is explained by the model (R-square = 0.054), which means that the large differences in ACT plasma levels between patients cannot be explained by the variables included in the model.

### ACT levels and mortality

**Fig. 2:**
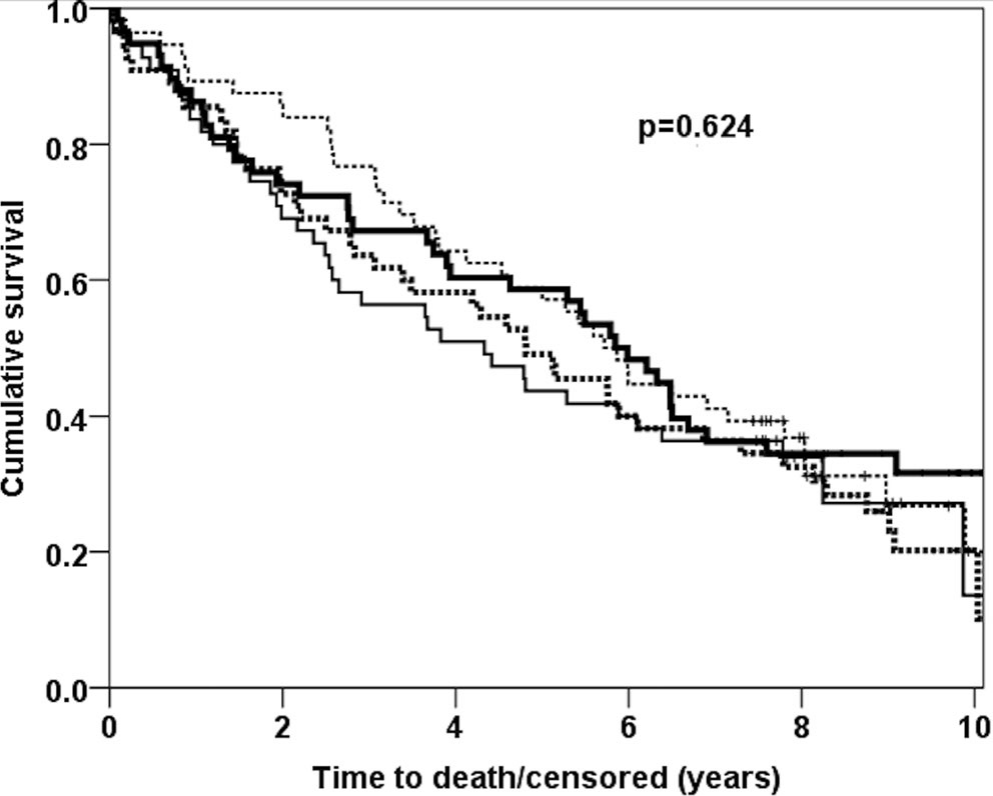
Kaplan-Meier curves for alpha-1-antichymotrypsin (ACT) levels. ACT levels were categorised in quartiles for presentation purposes. Quartile 1 (*solid bold*) consists of ACT levels between 93 and 297 μg/ml, quartile 2 (*dashed bold*) 297–434, quartile 3 (*dashed*) 434–687, quartile 4 (*solid*) 687–4545. ACT levels showed a non-significant effect on mortality

The mean and median survival time was 5.5 and 5.3 years, respectively. In total, 159 (71 %) patients died. Cox-proportional hazard regression models showed that ACT plasma levels (mg/ml) were not significantly related to long-term mortality (crude HR = 1.03, 95 % CI 0.75–1.41, *p* = 0.871; adjusted HR = 1.12, 95 % CI 0.78–1.60, *p* = 0.552). HR present the effect of ACT per 1000 μg/ml = 1 mg/ml. This non-significant effect of ACT was also confirmed by the Kaplan-Meier curves presented in Fig. 2, where circulating ACT was divided into quartiles and presented as Kaplan-Meier curves.

## Discussion

In the present study, ACT levels were significantly elevated in chronic HF patients in comparison with healthy controls and demonstrated that a single measurement of plasma ACT is not an independent risk factor for long-term mortality in these patients.

Multiple microarray analyses have been conducted to screen the gene expression profile of the failing myocardium from patients with dilated cardiomyopathy and suggested elevated ACT expression in the failing heart [13–16]. These findings were corroborated by our previous work in a small panel of severe end-stage HF patients who demonstrated profound elevated ACT levels in heart tissue as well as plasma at the time of left ventricular assist device implantation. The present study aimed to verify ACT up-regulation in a larger cohort of chronic HF patients.

ACT seems to be involved in the pathophysiology of HF. The exact role of ACT in HF is unknown, but several effects on the cardiovascular system have been postulated. ACT is a serine protease inhibitor, mainly of cathepsin G [17]. By eliminating cathepsin G, ACT might prevent the degradation of connective tissue proteins [18] and the activation of the transforming growth factor pathway [19], with subsequently less cardiomyocyte necrosis, hypertrophy and fibrosis. Also, ACT is an acute-phase protein and induces tumour necrosis factor (TNF)-α and NF-κB [20]. Additionally, ACT is thought to be protective during ischaemia reperfusion by inhibiting neutrophil accumulation into the ischaemic-reperfused myocardium and by inactivating cytotoxic metabolites released from neutrophils [21].

The present study demonstrated that ACT plasma levels were elevated in chronic HF patients, suggesting that ACT might be useful as a diagnostic marker in HF. Nevertheless, the present Cox PH regression analyses demonstrated that ACT is not an independent risk factor for long-term mortality in these patients with severe chronic HF. Future studies with plasma samples taken at different time points and taken from patients with less severe HF are necessary to analyse the potential role of ACT as a prognostic marker in HF.

## Limitations

The number of patients after 10 years of follow-up was small. As a sensitivity analysis, data up to 5 years of follow-up were also analysed with Cox regression, and showed similar results. Only one random plasma sample per patient was available for the present study. As a result, the possible effect of a change in levels of ACT on mortality is unknown. In this study, mainly chronic HF patients with an older age and reduced ejection fraction (HFrEF) were included. Our results cannot be extrapolated to young patients and/or less severe forms of HFrEF, acute HF and HF with a preserved left ventricular systolic function (HFpEF). The main exclusion criteria, expected survival of less than 1 year and planned hospitalisation, may have caused a selection bias, since these patients are expected to have high ACT levels. The endpoint of the present study was all-cause mortality. Therefore, we are not informed about the relation between the novel marker and hospitalisations for HF or other serious events.